# MLG: a mixed local and global model for brain tumor classification

**DOI:** 10.3389/fnins.2025.1618514

**Published:** 2025-07-03

**Authors:** Wenna Chen, Xinghua Tan, Jincan Zhang, Ganqin Du, Qizhi Fu, Hongwei Jiang

**Affiliations:** ^1^The First Affiliated Hospital, and College of Clinical Medicine of Henan University of Science and Technology, Luoyang, China; ^2^College of Information Engineering, Henan University of Science and Technology, Luoyang, China

**Keywords:** classification of brain tumor, CNN, transformer, feature fusion, gated attention mechanism

## Abstract

**Introduction:**

Brain tumors seriously endanger human health. Therefore, accurately identifying the types of brain tumors and adopting corresponding treatment methods is of vital importance, which is of great significance for saving patients’ lives. The use of computer-aided systems (CAD) for the differentiation of brain tumors has proved to be a reliable scheme.

**Methods:**

In this study, a highly accurate Mixed Local and Global (MLG) model for brain tumor classification is proposed. Compared to prior approaches, the MLG model achieves effective integration of local and global features by employing a gated attention mechanism. The MLG model employs Convolutional Neural Networks (CNNs) to extract local features from images and utilizes the Transformer to capture global characteristics. This comprehensive scheme renders the MLG model highly proficient in the task of brain tumor classification. Specifically, the MLG model is primarily composed of the REMA Block and the Biformer Block, which are fused through a gated attention mechanism. The REMA Block serves to extract local features, effectively preventing information loss and enhancing feature expressiveness. Conversely, the Biformer Block is responsible for extracting global features, adaptively focusing on relevant sets of key tokens based on query positions, thereby minimizing attention to irrelevant information and further boosting model performance. The integration of features extracted by the REMA Block and the Biformer Block through the gated attention mechanism further enhances the representation ability of the features.

**Results:**

To validate the performance of the MLG model, two publicly available datasets, namely the Chen and Kaggle datasets, were utilized for testing. Experimental results revealed that the MLG model achieved accuracies of 99.02% and 97.24% on the Chen and Kaggle datasets, respectively, surpassing other state-of-the-art models. This result fully demonstrates the effectiveness and superiority of the MLG model in the task of brain tumor classification.

## 1 Introduction

Brain diseases most commonly manifest as brain tumors, which represent a severe health threat to the human body and necessitate early diagnosis and treatment (Lyu et al., 2024; Akter et al., 2024; Liu et al., 2023). The classification of brain tumors constitutes a significant area of research in medical imaging and artificial intelligence. Classification of brain tumors using Magnetic Resonance Imaging (MRI) is the main technique (Li and Zhou, 2025). This process is critical for accurate diagnosis, treatment planning, and prognosis assessment. Recently, Computer-Aided Detection and Diagnosis (CAD) systems have played a pivotal role in assisting medical professionals with the detection and classification of brain tumors. Traditional manual methods of brain tumor classification rely heavily on experienced specialists and are often time-consuming, labor-intensive, and inefficient (Sharma et al., 2024; Zhou et al., 2024). To address this issue, extensive research has been conducted into automatic classification techniques that can classify brain tumors from MRI, employing CAD technology for tumor classification from MRI, which exhibits high reliability due to its high accuracy.

Traditional machine learning often relies on manually designed features, which places high demands on the user’s domain knowledge and experience. The selection and construction of features are complex and time-consuming, having a crucial impact on model performance. When faced with complex, high-dimensional, or nonlinear problems, the generalization ability of traditional machine learning algorithms may be limited (Kaur and Mahajan, 2025). More crucially, when confronted with new, unseen data, their predictive performance may decline, affecting their practical utility (Mehnatkesh et al., 2023; Pandiselvi and Maheswaran, 2019). In contrast, deep learning possesses stronger data representation capabilities, able to automatically learn high-level abstract representations of data, significantly enhancing the performance and effectiveness of machine learning. Deep learning models are not only highly complex but also capable of handling more complex tasks and larger datasets. Consequently, deep learning has found widespread application in the field of medical imaging, providing powerful support for disease diagnosis and treatment (Kshatri and Singh, 2023; Mazurowski et al., 2023; Mukadam and Patil, 2024; Yu et al., 2022).

Convolutional Neural Networks (CNNs), as a type of deep learning algorithm, have demonstrated remarkable prowess in the field of image processing, thanks to their unique advantages. The CNNs not only accept input images, but also adeptly assign varying degrees of importance to different elements or objects within those images through learnable weights and biases, enabling effective differentiation among them. Compared to other classification algorithms, the CNNs significantly reduce the need for preprocessing, greatly enhancing ease of use. In earlier image processing, filters were typically manually designed. However, CNNs can automatically learn these filters or features during training. Consequently, CNNs have seen widespread application in fields such as medical image analysis. Cao et al. (2024) introduced a Multi-branch Spectral Channel Attention Network (MbsCANet) for breast cancer classification. By extracting features in the frequency domain and applying attention mechanisms to the backbone network, MbsCANet achieves more precise feature extraction and classification, thereby not only improving classification accuracy but also providing robust support for early diagnosis and treatment of breast cancer. Regarding retinal disease classification, Peng et al. (2024) proposed a multi-scale-denoising residual convolutional network (MS-DRCN) model. This model integrates the strengths of Deep Residual Network (ResNet) along with multiscale processing and feature fusion techniques. Aimed at enhancing the accuracy and robustness of Optical Coherence Tomography (OCT) image classification, MS-DRCN offers an effective tool for precise diagnosis of retinal diseases. Moreover, SkinLesNet, a deep learning model specifically designed for skin lesion classification, is built upon a CNN architecture that has undergone meticulous design and optimization (Azeem et al., 2024). Through a series of CNNs, it progressively extracts image features, enabling in-depth understanding and analysis of lesion images. This structure enables the model to precisely capture subtle differences and key features within the images, significantly boosting classification accuracy and reliability. As a result, it provides crucial assistance in the early detection and treatment of skin lesions.

The Transformer, an attention mechanism originating from the field of natural language processing, has demonstrated remarkable performance in computer vision. Its advantages over CNNs are particularly evident in handling long-distance dependencies and global contextual information in images (Liu et al., 2021b; Yan et al., 2023; Huang S. K. et al., 2024). Bofan Song et al. (Song et al., 2024) utilized Vision Transformer (ViT) and Swin Transformer (SwinT) for the classification of oral cancer images. In the literature (Huang L. et al., 2024), Swin-residual transformer (SRT), was proposed for thyroid ultrasound image classification. The SRT model introduces residual blocks and triplet loss into the SwinT structure, aiming to improve sensitivity to both global and local features of thyroid nodules and better identify subtle feature differences. Additionally, Chincholi and Koestler (2024) designed a model combining ViT and Detection Transformer architectures for glaucoma detection. As the application of Transformers in disease detection continues to grow, researchers have begun exploring the integration of CNNs with Transformers to simultaneously extract local and global features. For instance, Fang et al. (2024) employed CNNs to extract local features while utilizing ViT for global feature extraction, designing a deep integrated feature fusion module for feature aggregation. Yan et al. (2023) developed the Transformer based High Resolution Network (TransHRNet) for brain tumor segmentation. TransHRNet initially used CNNs as an encoder for image preprocessing, followed by feeding the extracted features from the CNNs into an Effective Transformer (EffTrans) module, and finally generating segmentation results through a CNNs decoder. Notably, EffTrans incorporates Group Linear Transformations (GLTs) with an expansion-reduction strategy and spatial-reduction attention (SRA) layers, significantly reducing the computational burden and memory consumption of the Transformer.

The classification of brain tumors poses a highly challenging task in computer vision. These tumors vary significantly in size, shape, and location within the brain, and their categorization depends not only on the characteristics of the lesion itself but also on the surrounding tissue environment (ThamilSelvi et al., 2025; Verma and Yadav, 2025). Furthermore, the diversity and spatial distribution of brain tumors underscore the importance of utilizing both local and global features. In response to these challenges, the Mixed Local and Global (MLG) model is introduced. The uniqueness of the MLG model lies in its utilization of two advanced feature extraction methods. On one hand, Residual Efficient Multi-scale Attention (REMA) block is designed to extract local fine-grained features. On the other hand, the Bi-Level transformer (Biformer) block is used to capture the global context features. The REMA module integrates two layers of convolution and an Efficient Multi-scale Attention (EMA) component (Ouyang et al., 2023), which are interconnected through residual connections. This classical residual connection design ensures that gradients can propagate more effectively throughout the network during training, thereby mitigating gradient vanishing issues (He et al., 2016; Shafiq and Gu, 2022). Channel attention and spatial attention mechanisms have proven to be highly effective in generating more discriminative feature representations (Hu et al., 2018; Woo et al., 2018; Yu et al., 2023). In this block, EMA enhances both spatial and channel-wise features and achieves the ability to capture feature information across different scales by constructing parallel subnetwork structures operating at multiple resolutions. The core of Biformer is its Bi-Level Routing Attention (BRA), which facilitates dynamic and query-based content-aware sparse attention allocation while circumventing the high computational cost of full-space attention. Biformer realizes this pattern by introducing the Bi-Level Routing Attention mechanism, where it first prunes irrelevant key-value pairs at a coarse-grained region level, and subsequently conducts fine-grained token-to-token attention computations only within the selected candidate regions (Zhu et al., 2023). The integration of features from REMA and Biformer via gated attention mechanisms further refines these features, enhancing model performance. To validate the efficacy of the MLG model, two publicly available brain tumor datasets were utilized for experimental evaluation. Experimental results demonstrated that the proposed model outperforms other existing advanced models in terms of performance. In summary, the main contributions of this paper are as follows:

• Development of a brain tumor classification model that integrates both local and global features.
• The innovative application of the REMA module to extract local features and the use of Biformer for capturing global features, with both being effectively fused through a gated attention mechanism.
• Validation of the proposed model on two open datasets, achieving superior results compared to the current state-of-the-art performance.

## 2 Related work

The application of deep learning techniques in medical image analysis is becoming increasingly popular, particularly in the study of brain tumor classification, where it has demonstrated significant value. In recent years, research efforts on brain tumor classification tasks have continued to deepen, and these studies can be broadly categorized into two camps: one is the CNN-based approach, and the other is the emerging strategy based on the Transformer architecture.

### 2.1 CNN in brain tumor classification

The CNN has been widely used in brain tumor classification tasks. In the task of brain tumor classification, CNNs have been widely employed. Kang et al. (2021) adopted a transfer learning-based framework using a pre-trained deep CNN to extract deep features from MRI data. By fusing features obtained from different levels of the network and integrating them with multiple machine learning classifiers, this method achieved significant results. Alanazi et al. (2022) proposed a 22-layer CNN model, which was initially trained on a binary brain tumor dataset. Subsequently, with the help of transfer learning technique, the model weight was utilized for multi-class data, resulting in promising outcomes. Saurav et al. (2023) designed an Attention-Guided Convolutional Neural Network (AG-CNN) specifically tailored for brain tumor classification tasks. The network incorporates an internal channel attention module, which aids in focusing on processing image regions relevant to tumors, thereby facilitating effective feature extraction and classification. Alturki et al. (2023) proposed an optimization scheme for brain tumor classification performance. The CNNs were utilized to extract deep features from raw brain tumor MRI data and two classification algorithms including logistic regression (LR) and stochastic gradient descent (SGD) were incorporated into a voting ensemble classifier. By inputting these deep features into the ensemble classifier, the model achieved accurate classification of brain tumors. Hossain et al. (2023) conducted a study implementing transfer learning to investigate the performance of various models, including VGG16, InceptionV3, and ResNet50, inceptionResNetv2, Xception, for brain tumor classification. Ultimately, three best performing models were chosen to be used to construct an ensemble model, which was named IVX16. Sachdeva et al. (2024) evaluated multiple pre-trained models such as ResNet50, DenseNet121, EfficientNetB0, and EfficientNetV2L, et al., by incorporating Dropout layers, global average pooling layers, and tuning hyperparameters to enhance model performance. The results show that EfficientNetB0 model achieved a higher classification accuracy.

### 2.2 Transformer in brain tumor classification

Transformer has also been applied in brain tumor classification tasks. Ferdous et al. (2023) proposed a Linear Complexity Data-Efficient Image Transformer (LCDEiT) framework based on a teacher-student mechanism specifically designed for tumor classification from brain MRI images. In the teacher model component, gated pooling techniques were employed to optimize the feature extraction efficiency of CNNs. The pre-trained teacher model was able to extract crucial knowledge pertinent to the tumor classification task. On the other hand, the student model introduced an image transformer equipped with an external attention mechanism, which leveraged the knowledge acquired from the teacher model for tumor classification in brain MRI. In paper, Asiri et al. (2024) proposed an innovative and robust method based on the SwinT architecture, aiming to improve the accuracy of brain tumor image classification. This method integrated complex preprocessing procedure, sophisticated feature extraction techniques, and a thorough classification system, enabling the SwinT model to effectively analyze and discriminate various types of brain tumors. Wang et al. (2024) employed a pre-trained ViT as the backbone for their brain tumor classification model, named as RanMerFormer. Additionally, to enhance the computational efficiency of the ViT backbone, a Token Merging Algorithm (TMA) was used. Instead of using a traditional linear classification head, Random Vector Functional Link (RVFL) networks were utilized. Poornam and Angelina (2024) proposed the ViT with Attention and Linear Transformation module (VITALT) for brain tumor detection and classification. VITALT primarily consists of a ViT, a Split bidirectional feature pyramid network (S-BiFPN), and a linear transformation module (LTM). ViT was used to capture global and local features, while S-BiFPN fusions the features extracted by ViT. The LTM enhanced the model’s linear expressive ability. In paper (Şahin et al., 2024), the Bayesian Multi-Objective (BMO) optimization method was employed to optimize the hyperparameters of the ViT network in order to improve its performance in brain tumor classification tasks. Gade et al. (2024) proposed the Lite Swin Transformer (OLiST) model for brain tumor detection. This model combined the Lite Swin Transformer’s ability to capture global features with the advantage of CNNs in extracting local features. By fusing the features extracted by both, the model leveraged the strengths of both approaches.

In summary, the use of CNNs and Transformers have been used in brain tumor classification tasks with excellent performance. CNNs have the advantage of extracting local features of images, while Transformers have the advantage of exploiting global features of images. Therefore, this paper innovatively introduces a hybrid model, MLG, which effectively integrates the respective strengths of CNNs and Transformers, thus significantly enhancing the performance of brain tumor classification tasks.

## 3 Materials and methods

In this section, the datasets used and the proposed model are described in detail.

### 3.1 Datasets and preprocessing

In this study, two widely used public datasets, namely the Chen dataset and the Kaggle dataset, were adopted. The Chen dataset, provided by Cheng et al. (2015), primarily focuses on three types of brain tumors: gliomas, meningiomas, and pituitary tumors. Comprising a total of 3,064 images, this dataset offers a rich resource for our in-depth research and analysis. On the other hand, the Kaggle dataset is a meticulously compiled and shared public dataset by Bhuvaji et al. (2020). This dataset encompasses four categories of images: glioma tumors, meningioma tumors, pituitary tumors, and normal brain tissues, totaling 3,264 images. For efficient model training and testing, the two datasets were randomly divided into a training set and a test set. Specifically, 80% of the data was allocated to the training set for model training and optimization, while the remaining 20% was designated as the testing set for evaluating the model’s performance. Detailed statistics on the number of images in each dataset are presented in Table 1.

**TABLE 1:** Details of the datasets.

Dataset name	Classes	Number of each class	Total image count
Chen	Glioma	1,426	3,064
	Meningioma	708	
	Pituitary tumor	930	
Kaggle	Glioma	826	3,264
	Meningioma	822	
	Pituitary tumor	827	
	No tumor	395	

A simple and efficient data preprocessing method is used in the preprocessing phase of the dataset. In the experimental process, to preserve the integrity of image content and stability of features, all images were uniformly resized to 224 × 224 × 3 pixels. This resizing not only helps maintain the spatial structure and information integrity of the images but also significantly reduces computational burden during network training, thereby enhancing training efficiency. Additionally, normalization was performed, which is a standard preprocessing step in deep learning. This aims to mitigate differences in brightness, contrast, and other attributes among images, enabling the model to focus more acutely on learning the inherent features of the images. For medical images, acquiring a large volume of such data can be challenging (Dhar et al., 2023). Given that deep neural networks typically require substantial amounts of data for training, and considering the relatively limited scale of the datasets utilized in this study, data augmentation strategies were employed to alleviate overfitting concerns. Specifically, random rotation and random horizontal flipping techniques were utilized, both of which effectively enhance dataset diversity without introducing additional noise, thereby improving the model’s generalization capability.

### 3.2 Mixed local and global model

**FIGURE 1:**
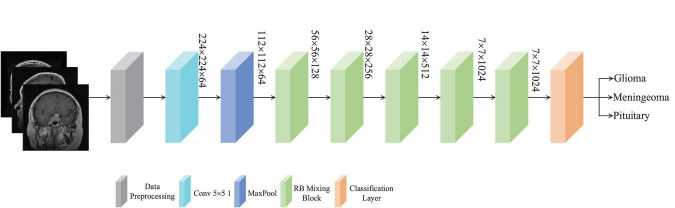
Proposed brain tumor classification system.

**FIGURE 2:**
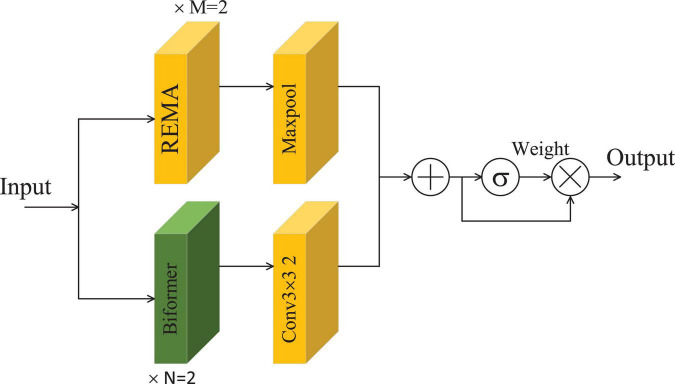
The RB Mixing Block structure.

In this section, details of the proposed model are provided. The architecture of the MLG model, which combines both local and global components, is depicted in Figure 1. Initially, brain tumor images undergo preprocessing before being fed into a convolutional layer with a kernel size of 5 × 5 and a stride of 1, designed to enlarge the receptive field. Subsequently, a max pooling layer is applied for downsampling and dimensionality reduction of the extracted features. And then, the features are further processed through five REMA and Biformer (RB) Mixing Blocks to refine the extraction of characteristics specific to brain tumor images. Finally, the resulting features are classified accordingly. The structure of the RB Mixing Block is illustrated in Figure 2.

Figure 2 presents the structure of the RB Mixing Block, primarily consisting of REMA and Biformer units. The REMA unit is designed to extract local features from the images, while the Biformer unit focuses on extracting global features. After combining the features derived from these two modules, a gating mechanism adjusts the weights of the fused features to better suit the task of brain tumor classification, thereby enhancing the model’s classification performance. Here, M denotes the number of REMA convolution modules used and N denotes the number of Biformer modules used, *M* = *N* = 2. REMA utilizes max pooling for downsampling, aiming to broaden the receptive field of the module. On the other hand, Biformer employs convolutions with a stride of 2 for downsampling, intending to derive higher-level feature representations. Subsequently, the features extracted by both REMA and Biformer are merged and subjected to processing by the gating mechanism. Then, the adjusted features are multiplied with the original ones to modulate their significance in influencing the model’s overall performance, effectively filtering out a set of features that have a more substantial impact on the model’s classification results. The output of the RB Mixing module can be expressed as:


(1) $${\text{out}}_{R\,B}=s\,i\,g\,m\,o\,i\,d\,\left(f_{\text{REMA}}+f_{\text{Biformer}}\right)\times \left(f_{\text{REMA}}+f_{\text{Biformer}}\right)$$

where*, f_*REMA*_* and *f*_*Biformer*_ represent the features extracted by the modules REMA and Biformer, respectively.

In order to present the structure and parameter characteristics of the REMA module and the Biformer module more clearly. We have detailed the number of parameters, input dimensions and output dimensions of these two modules in Table 2.

**TABLE 2:** Parameters and dimension information of the REMA block and the Biformer block.

Block	Input size	Output size	No. of parameters
REMA	112 × 112 × 64	112 × 112 × 64	74,160
Biformer	112 ×112 × 64	112 × 112 × 64	10,4576

The structure and computational complexity of the REMA block and the Biformer block in the MLG model can be understood more specifically through Table 2.

### 3.3 REMA Block

**FIGURE 3:**
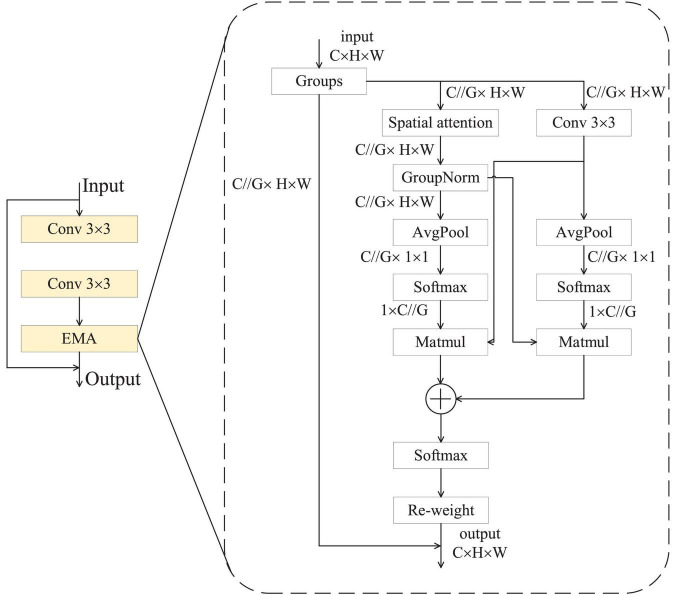
REMA structure.

The structure of the REMA block is depicted in Figure 3. This module consists of two convolutional layers and an EMA unit, interconnected via residual connections to facilitate information fusion and propagation. This design aims to enhance the model’s representation learning capacity while alleviating the gradient vanishing problem often encountered in deep networks. By incorporating the EMA unit (Ouyang et al., 2023), the REMA block is better equipped to capture inherent data features, thereby boosting the model’s performance. The core idea of the EMA module is to group the channel dimensions into multiple sub-features and ensure good distribution of spatial semantic features within each feature group. This method not only preserves information in each channel but also reduces computational overhead. Specifically, the EMA module recalibrates the channel weights of each parallel branch using global information encoding. Moreover, the output features from the two parallel branches are aggregated through cross-dimensional interaction methods, further enhancing the representational power of the features. Inside the EMA module, there are three parallel paths designed to extract attention weight descriptors for the grouped feature maps. Two of these paths belong to the 1 × 1 branch, while the third one is part of the 3 × 3 branch. Within the 1 × 1 branch, two one-dimension global average pooling operations along two spatial directions are employed to encode channel attention. In contrast, the 3 × 3 branch uses a single 3 × 3 convolutional kernel to capture multi-scale feature representations. The output of the REMA module can be mathematically represented as follows:


(2) out=E⁢M⁢A⁢(c⁢o⁢n⁢v⁢(c⁢o⁢n⁢v⁢(x)))+x

**FIGURE 4:**
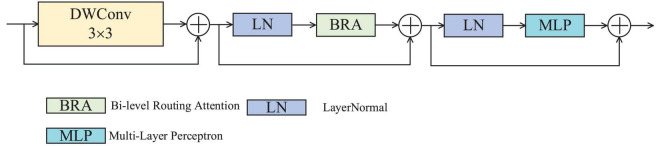
Biformer structure.

The structure of the Biformer Block is depicted in Figure 4. The core of the Biformer lies in its BRA, which consists of a deep convolution, two layers of Layer Normalization (LN), and a Multilayer Perceptron (MLP) interconnected through residual connections (Zhu et al., 2023).

The design principle of BRA revolves around dynamic, query-content based sparsity. Initially, irrelevant key-value pairs are filtered out at a coarse-grained regional level by constructing and pruning a directed graph representing region-level relationships. Subsequently, a fine-grained token-to-token attention mechanism is applied over the joint set of the remaining, or routed, regions to selectively focus on locally relevant information while bypassing globally unrelated data. In BRA process, given a two-dimensional input feature map X, it is partitioned into S × S non-overlapping regions, each containing a specific number of feature vectors. These region-based features undergo linear projections to generate query, key, and value tensors Q, K, V. An inter-region association matrix A^γ^ is then constructed by computing average query and key vectors across regions, with its elements indicating semantic relevance between pairs of regions. The critical step involves selecting the top k most related adjacent regions for each region based on this relevance measure, yielding a routing index matrix I^γ^ via row-wise top-k operations. Building upon this, the model applies fine-grained token-to-token attention. Specifically, for a query token originating from region i, it attends to all key-value pairs within the k routed regions indexed by I${}_{\left(i,1\right)}^{\gamma }$ through I${}_{\left(i,k\right)}^{\gamma }$. To efficiently execute this, despite these regions potentially being scattered throughout the feature map, the model first employs a gather operation to collect the key and value tensors from these regions, forming aggregated key and value sets K_*g*_ and V_*g*_. Finally, attention computation is performed using the gathered key and value tensors:


(3) $$O=s\,o\,f\,t\,\max\left(\frac{{\left(Q\,K_{g}\right)}^{T}}{\sqrt{C}}\right)\,V_{g}+L\,C\,E\,\left(V\right)$$

here, ${}_{\sqrt{c}}$ is usually a factor that scales the denominator in the formula for calculating the attention score in order to prevent the occurrence of over-concentration of weights and loss of gradients. *LCE (V)* represents local context enhancement, which is implemented by depth separable convolution to enhance local information.

### 3.4 Loss function

In classification tasks, the cross-entropy loss function is a commonly used loss function. Originating from the concepts of entropy and mutual information in information theory, it serves to quantify the discrepancy between two probability distributions. Specifically, when training neural networks, it is employed to measure the difference between the model’s predicted probability distribution and the true distribution of the observed data. For classification tasks, assuming the true label is y and the model predicted probability is q, the cross-entropy loss function can be expressed as:


(4) H⁢(y,q)=-∑iy⁢logi⁡(qi)

where, *y*_*i*_ represents the true label for the i-th category and q_*i*_ denotes the model predicted probability that the sample belongs to the i-th class.

## 4 Results

This section introduces the experimental setup, experimental results, and ablation experiments, collectively serving to comprehensively and rigorously substantiate the proposed model.

### 4.1 Experimental apparatus

A PyTorch implementation is performed for the model proposed by us, while experiments were carried out on a Windows 11 system equipped with a 12GB RTX 4070 GPU and an Intel i5-13400F processor. The Adam optimizer was utilized, with the initial learning rate set at 0.0001, the batch size fixed at 16, and the number of epochs specified as 50. In our experiments, early stopping was utilized to prevent overfitting. Detailed information about the parameters can be found in Table 3.

**TABLE 3:** Training Hyper-parameter values of proposed network.

Parameters	Value
Initial learning rate	0.0001
Batch size	16
Optimizer	Adam
Number of epoch	50
Learning rate decays	0.1

### 4.2 Evaluation metrics

In the experiments, the accuracy, recall, precision, and F1-score were employed as evaluation metrics, with their respective calculation methods presented in Formulas (5–8). The accuracy is one of the most commonly used evaluation metrics in classification problems, representing the proportion of correctly classified samples out of the total number of samples. The recall, focuses on the ability of the model to correctly identify positive samples, which refers to the ratio of true positives (correctly identified positive instances) to all actual positive instances in the dataset. The precision measures the proportion of instances predicted by the model as positive that are truly positive, that is, the ratio of true positives to all instances predicted as positive. The F1-score, being the harmonic mean of precision and recall, integrates the performance of both precision and recall, offering a more comprehensive assessment of the model’s performance (Zulfiqar et al., 2023; Zebari et al., 2024). When both precision and recall are high, the F1-score will also be high, and conversely, when either of these values is low, so will the F1-score. This implies that a high F1-score indicates strong overall performance in terms of both accurately identifying true positives and minimizing false predictions.


(5) $$\mathrm{Accuracy}=\frac{T\,N+T\,P}{T\,N+T\,P+F\,N+F\,P}$$

(6) $$R\,e\,c\,a\,l\,l=\frac{T\,P}{T\,P+F\,N}$$

(7) $$P\,r\,e\,c\,i\,s\,i\,o\,n=\frac{T\,P}{T\,P+F\,P}$$

(8) $$F\,1-s\,c\,o\,r\,e=\frac{2\,T\,P}{2\,T\,P+F\,P+F\,N}$$

### 4.3 The results of the experiment

**FIGURE 5:**
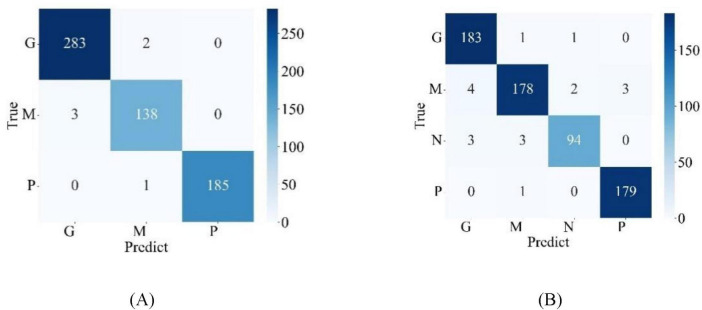
Confusion matrix for model classification results **(A)** Chen dataset **(B)** Kaggle dataset.

Figure 5 illustrates the confusion matrices for the classification results of the model on the test sets of two publicly available datasets, where G, M, and P stand for glioma, meningioma, and pituitary adenoma, respectively, and N stands for normal state, indicating the absence of brain tumor. From the confusion matrices, detailed classification performance metrics for the model were calculated according to Formulas (5–8) and summarized in Table 4. From Table 4, it is evident that, on the test set of the Chen dataset, the average performance metrics for model MLG include a recall of 98.88%, precision of 98.94%, F1-score of 98.91%, and accuracy of 99.02%. On the Kaggle dataset test set, MLG corresponding metrics are 96.89% for recall, 97.21% for precision, 96.89% for F1-score, and 97.24% for accuracy. These indicators demonstrate that across both the Chen and Kaggle datasets, the MLG model exhibits outstanding classification performance, which further validates the effectiveness and generalization capabilities of the MLG model, enabling it to achieve satisfactory performance in brain tumor classification tasks on diverse datasets.

**TABLE 4:** Detailed values of metrics for the proposed model.

Dataset	Tumor type	Recall (%)	Precision (%)	F1-score (%)	Accuracy (%)
Chen	Glioma	99.30	98.95	99.12	99.02
	Meningioma	97.87	97.87	97.87	
	Pituitary	99.46	1.00	99.73	
	**Average**	**98.88**	**98.94**	**98.91**	
Kaggle	Glioma	98.92	96.32	97.60	97.24
	Meningioma	95.19	97.27	96.22	
	No Tumor	94.00	96.91	95.43	
	Pituitary	99.44	98.35	98.90	
	**Average**	**96.89**	**97.21**	**96.89**	

### 4.4 Ablation study

In Section 4.3, performance metrics for the classification results of the proposed model are presented. To further confirm the validity of the proposed model, an ablation study was performed. In this study, different combinations of modules are explored within the framework of the model. This process allows for a meticulous examination of each component’s contribution to the overall performance, thereby providing deeper insights into the effectiveness and robustness of the proposed model architecture.

**FIGURE 6:**
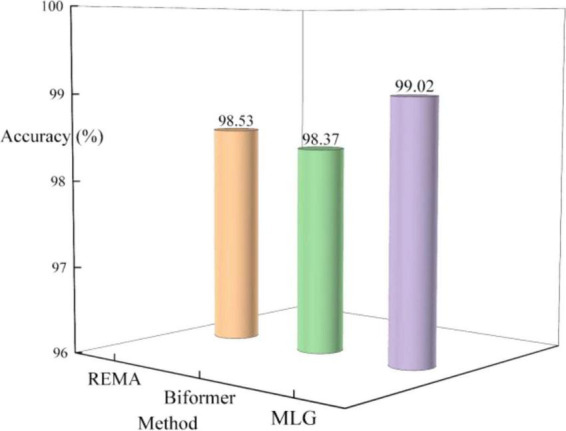
Classification results by REMA and Biformer.

In the first part of the study, brain tumor classification was conducted separately using REMA and Biformer independently. Figure 6 presents the testing results of various models in the Chen dataset during the ablation experiment. The accuracies achieved by REMA and Biformer are 98.53 and 98.37%, respectively, both lower than the 99.02% accuracy obtained by MLG. Upon conducting a detailed analysis of the ablation experiment results, it becomes clear that the integration of the strengths of both the REMA and Biformer modules within the MLG model effectively boosts the accuracy rate in brain tumor classification.

**FIGURE 7:**
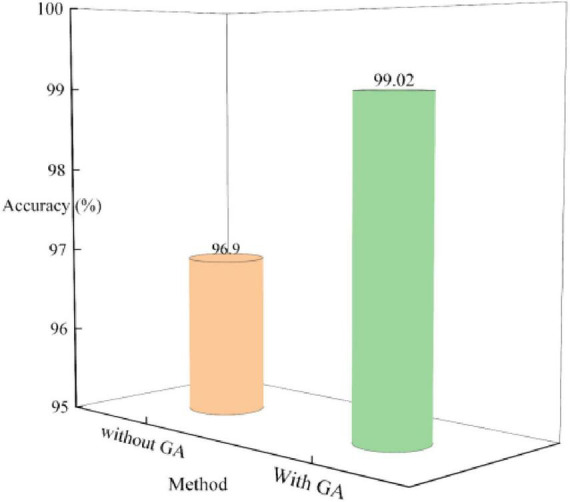
Effects of gating attention mechanism on MLG.

In the second part of the study, the performance of the MLG model upon incorporating the gated attention mechanism was meticulously examined. The gated attention mechanism plays a pivotal role within the model, serving to regulate the flow of information by deciding which pieces of information should be emphasized and which should be disregarded. By means of gating, the attention mechanism assigns weights to information based on its importance, thereby enhancing the model performance by focusing on crucial features. Figure 7 shows the performance of the model with and without the gated attention mechanism. Where, GA stands for Gated Attention. It can be observed that when the model does not include the gated attention, its performance lags behind the version with the gated attention mechanism by 2.12%. The results strongly demonstrate the effectiveness of the gated attention in improving the performance of the model.

**FIGURE 8:**
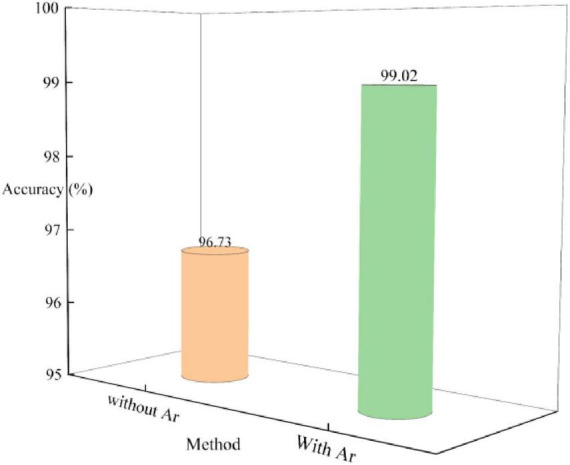
Impact of data augmentation on MLG.

In the third segment of the investigation, the impact of data augmentation on the MLG model was thoroughly explored, particularly in scenarios involving small sample datasets. Data augmentation is a critical technique that can significantly enhance a model generalization capability while mitigating overfitting issues. In this work, two prevalent data augmentation strategies were employed: random rotation and random flipping. Figure 8 provides a detailed account of the model accuracy rates on both the training and test sets of the Chen dataset when data augmentation is applied. Ar stands for data augmentation. From the figure, it is evident that with data augmentation, the training and test set accuracies reach 99.96 and 99.02%, respectively. In contrast, without data augmentation, while the accuracy on the training set reached 100%, the accuracy on the test set notably decreased to 96.73%. This comparative outcome vividly demonstrates that data augmentation has a pronounced effect on improving model performance.

## 5 Discussion

According to the data in Table 4, the MLG model achieves impressive accuracies of 99.02% on the Chen dataset and 97.24% on the Kaggle dataset, which attest to its effectiveness and satisfactory performance. Moreover, through ablation studies, the superiority of the MLG model was further substantiated, emphasizing the significant improvements gained by fusing the REMA and Biformer modules via the gated attention mechanism, rather than merely adding them together. Additionally, the application of data augmentation has led to noticeable performance enhancements, further bolstering the model generalization capabilities.

Beyond internal validation, the proposed model was also compared against other advanced methods utilizing the same datasets. Table 5 clearly outlines these comparative results. On the Chen dataset, the MLG model outperforms the current best-performing model, Multimodal-CNN Model (Maqsood et al., 2022), by 0.1% in accuracy. Similarly, on the Kaggle dataset, the MLG model surpasses the previously best-reported model IVX16 (Hossain et al., 2023) by an accuracy margin of 0.3%. When juxtaposed against methodologies outlined in literature sources paper (Alanazi et al., 2022)and paper (Saurav et al., 2023), the MLG model consistently demonstrates higher performance on both the Chen and Kaggle datasets. Precisely, on the Chen dataset, MLG accuracy exceeds that of paper (Alanazi et al., 2022) by 2.13% and that of paper (Saurav et al., 2023) by 1.79%. On the Kaggle dataset, MLG accuracy advantage over paper (Alanazi et al., 2022) is 1.49%, while over (Saurav et al., 2023) it is 1.53%. These comparative results serve as compelling evidence of the MLG model superior performance in the task of brain tumor classification, reinforcing its potential applicability in real-world scenarios.

**TABLE 5:** Compare with advanced methods on datasets Chen and Kaggle.

Method category	References	Method	Dataset	Accuracy (%)
CNN	Sachdeva et al. (2024)	Transfer learning	Kaggle	96.25
	Jun and Liyuan (2022)	Attention-Guided	Chen	98.61
	Maqsood et al. (2022)	Multimodal-CNN Model	Chen	98.92b
	Alanazi et al. (2022)	22-layer CNN	Chen	96.89
			Kaggle	95.75
	Saurav et al. (2023)	AG-CNN	Chen	97.23
			Kaggle	95.71
Transformer	Wang et al. (2024)	RanMerFormer	Chen	98.86
	Şahin et al. (2024)	BMO	Chen	98.09
	Hossain et al. (2023)	IVX16	Kaggle	96.94
	Anaya-Isaza et al. (2023)	Cross-Transformer	Chen	97.22
	Dosovitskiy et al. (2021)	Vision Transformer	Chen	97.39
			Kaggle	95.88
	Liu et al. (2021a)	Swin Transformer	Chen	98.69
			Kaggle	97.10
CNN+ transformer	Ferdous et al. (2023)	LCDEiT	Chen	98.11
	Chen et al. (2025)	EnSLDe	Chen	98.69
	Proposed model	MLG	Chen	99.02
			Kaggle	97.24

**FIGURE 9:**
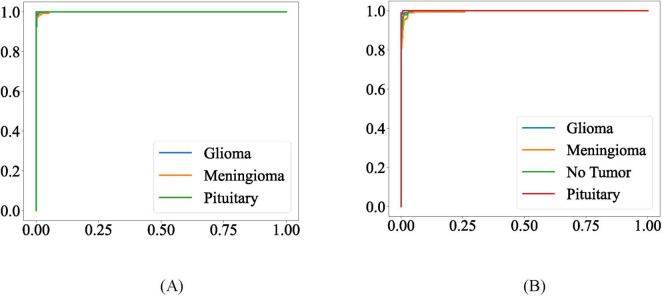
ROC curves for the proposed model on **(A)** Chen dataset **(B)** Kaggle dataset.

The Receiver Operating Characteristic Curve (ROC Curve) is a widely used visualization tool in statistics, machine learning, medical diagnostics, and other fields that require categorical judgments for evaluating the performance of classification models. It graphically illustrates the trade-off relationship between the true positive rate (TPR) and false positive rate (FPR) of the model under different threshold conditions. The area under curve (AUC), indicates better model performance when its value is larger. Typically, the closer the curve is to the upper left corner (with higher TPR and lower FPR), the better the model performance. The ROC curves of the model on the two datasets are shown in Figure 9. It can be observed that the ROC curves closely adhere to the upper left corner. On the Chen dataset, the AUC values of the MLG model for glioma, meningioma, and pituitary tumors are 0.9996, 0.9993, and 1.00, respectively. Meanwhile, on the Kaggle dataset, the AUC values of the MLG model for glioma, meningioma, normal tissue, and pituitary tumors are 0.9991, 0.9965, 0.9989, and 0.9999, respectively.

## 6 Conclusion

Brain tumors, constituting a severe health issue, pose a significant threat to people’s lives. Therefore, timely and accurate identification of brain tumor types, followed by appropriate treatment planning, is critical for patients. The advent of CAD technology has provided substantial support to doctors in diagnosing brain tumors. In this paper, a novel MLG brain tumor classification model is proposed, and the model skillfully integrates local features and global features, and provides a new solution for the classification of brain tumors. The core components of the MLG model are RMEA, Biformer and gated attention. The RMEA Block, through carefully designed convolutional structures, efficiently retains information across channels, emphasizing spatial and channel-wise features, thereby extracting richly informative local features. Conversely, the Biformer employs a unique BRA mechanism to dynamically and contextually select a subset of the most relevant key-value pairs for each query, optimizing the computational process. Meanwhile, BRA can capture remote dependencies across regions and even objects, providing powerful support for extracting global features. The MLG model uses a gated attention to selectively filter and fuse the local features extracted by the RMEA block with the global features extracted by the Biformer block. This significantly enhances the representation capability of the fused features, thereby improving the classification performance of the model. The integration of both local and global features enables the MLG model to exhibit outstanding performance in brain tumor classification tasks. Experimental results on two public datasets demonstrate that the MLG model achieves satisfactory performance across multiple metrics, including accuracy, precision, recall, and F1-score. Compared with existing advanced methods, the MLG model exhibits marked advantages, fully validating its effectiveness in practical applications. In future work, it is planned to continue exploring other methods of feature fusion first to further improve the performance of the MLG model. Secondly, the introduction of more refined feature detection methods will be explored, or they will be combined with other advanced attention mechanisms to enhance the selection ability for key areas. In addition, efforts will also be made to obtain data on other brain diseases, expand the application scope of the model, and provide more auxiliary diagnostic tools for the medical field.

## Data Availability

Publicly available datasets were analyzed in this study. This data can be found at: the datasets used are free and open. Dataset Chen from figshare (https://figshare.com/articles/dataset/brain_tumor_dataset/1512427). Dataset Kaggle from Kaggle (https://www.kaggle.com/datasets/sartajbhuvaji/brain-tumor-classification-mri).
